# From Pattern Recognition to Abstract Representation: Visual Search and Neural Mechanisms of Structured Pattern Processing in Skilled Basketball Players

**DOI:** 10.3390/jemr19050116

**Published:** 2026-10-08

**Authors:** Na Xu, Xiaoqin Wang, Xiuying Miao, Juntong Song, Xin Zhang, Pengfei Ren, Yue Zhang, Lizhong Chi

**Affiliations:** 1 School of Psychology, University of International Relations, Beijing 100091, China; xuna@uir.edu.cn; 2 Shanghai University of Finance and Economics Zhejiang College, Jinhua 321000, China; 3 School of Physical Education, Hanshan Normal University, Chaozhou 521041, China; 4 China Basketball College, Beijing Sport University, Beijing 100084, China; 5 School of Psychology, Beijing Sport University, Beijing 100084, China; 6 College of Sports and Health, Shandong Sport University, Jinan 250102, China; 7 School of Physical Education, Central China Normal University, Wuhan 430079, China

**Keywords:** skilled basketball players, structured pattern, pattern recognition, abstract representation, transformer

## Abstract

Objective: This study employed eye-tracking and ERP to investigate structured pattern processing in basketball players of different skill levels. Methods: A skill-level comparison (skilled vs. less-skilled) was adopted. Participants viewed static point-light displays depicting basketball half-court configurations, with structured (typical tactical formations) and unstructured (random positions) conditions randomly presented. Participants predicted offensive actions while behavioral, eye-tracking, and ERP data were simultaneously recorded. Results: Skilled players showed higher decision accuracy than less-skilled players under structured conditions but not under unstructured conditions, whereas they responded significantly faster under both conditions, with a larger group difference under structured conditions. For pupil diameter, skilled players showed smaller pupil diameters than less-skilled players under both conditions, while within the skilled group pupil diameter was larger under structured than unstructured conditions. Eye-tracking revealed fewer total fixations and a distinct gaze distribution in skilled players—more fixations on opponents and the ball, fewer on open space—along with larger pupil diameters under structured conditions, suggesting enhanced cognitive engagement. ERP data showed that skilled players exhibited larger P300 and LNC amplitudes under structured conditions, reflecting enhanced semantic mapping and schema-based retrieval. Conclusions: These findings are consistent with a theoretical perspective in which expertise may involve a shift from “pattern recognition” toward “abstract representation,” rather than a reduction in neural activity. However, abstract representation, neural information density, and semantic encoding were not directly measured in the present study. The Transformer architecture is therefore offered as a conceptual analogy—its multi-head self-attention corresponds to skilled players’ hierarchical visual sampling, its encoder to semantic mapping (P300 enhancement), and its memory integration to schema-based retrieval (LNC enhancement). These findings provide a basis for structured situational training in sport decision-making.

## 1. Introduction

Why can skilled players process structured patterns more efficiently? How do humans learn to become skilled players? These two questions constitute the core issues in understanding the development of human skilled players and are the fundamental questions that this study seeks to answer.

In competitive sports, fast and high-quality decision-making is the foundation of skilled-player performance, requiring athletes to process complex spatiotemporal patterns in real time [1,2]. Pattern recognition plays a central role in this process, enabling athletes to extract structural features—such as player positioning, offensive and defensive formations, and movement trajectories—thereby simplifying information processing and optimizing decision-making efficiency [3,4,5]. In basketball, players must integrate multiple sources of information from teammates, opponents, the ball, and court dynamics while adjusting their focus to key situational cues [6]. These structured patterns were operationalized in this study as quantifiable player spatial positions (e.g., 2–3 zone defense, 1-3-1 set) in basketball half-court offensive and defensive scenarios, facilitating anticipation and decision-making in high-intensity, fast-paced game environments [7].

The abstract representation hypothesis [8] proposes that the cognitive advantage of skilled sports players stems from their ability to form abstract representations of professional knowledge and skills. Abstract knowledge provides necessary constraints for learning [9], and the schemas stored in skilled players’ long-term memory are more abstract and hierarchical than those of less-skilled players, thereby supporting more efficient pattern recognition and decision transfer. This view is highly consistent with long-term working memory theory [10] and deliberate practice theory [11]. However, to test this hypothesis, three more operational sub-questions must be further addressed: First, how do skilled players selectively attend to key structured information in dynamic scenes? Second, how do skilled players semantically map visual patterns into tactical concepts that guide decision-making? Third, how do skilled players achieve optimal allocation of neural resources through memory integration?

Regarding the first question—selective attention and visual search strategies—existing eye-tracking studies have revealed significant differences between skilled sports players and less-skilled players. Research indicates that experienced athletes tend to focus on global information during visual orientation, exhibiting fewer fixations and shorter fixation durations compared to less-skilled players [12]. However, other studies have reported that skilled athletes sometimes exhibit more fixations under certain conditions [13]. This discrepancy may stem from differences in task constraints [14] and response types [15]. Synthesizing these findings, a more consistent observation is that skilled players possess an efficient “global–local” visual search pattern: they quickly extract scene structure (e.g., formation recognition) through fewer but more strategic first fixations, then shift focus to verify local details (e.g., player movements or passing options). This study defines this pattern as a hierarchical visual sampling strategy and hypothesizes that this strategy is the core mechanism through which skilled players achieve optimized selective attention.

Regarding the second question—semantic mapping and behavioral decision advantage—a large body of research has demonstrated that skilled players exhibit faster decision speeds and higher accuracy when processing structured game patterns [16]. However, a key contradiction remains unresolved: does this advantage stem from enhanced perceptual encoding (i.e., skilled players extract and organize key information more efficiently) or from automated response selection (i.e., familiar patterns trigger pre-learned responses with minimal cognitive effort)? Traditional single-dimensional behavioral metrics such as reaction time and accuracy cannot distinguish between these two possibilities [5,17]. This study proposes that the core of this decision advantage lies in semantic mapping ability: skilled players can rapidly recognize positioning as tactical concepts (e.g., “pick-and-roll”), identify movement as tactical intentions (e.g., “fast break”), and perceive open spaces as passing opportunities. In other words, skilled players’ decision advantage is not merely a reduction in reaction time but is built upon efficient semantic encoding of structured patterns—a cognitive transition from “seeing” to “understanding.” This study hypothesizes that under structured conditions, skilled basketball players make faster and more accurate decisions, reflecting their learned ability to efficiently recognize and semanticize structured patterns.

Regarding the third question—memory integration and neural resource optimization—ERP research provides a critical window into understanding the neural mechanisms of skilled performance. However, the literature on sport expertise ERP does not converge on a single pattern of P300 modulation. Some studies support the neural efficiency hypothesis, showing smaller P300 amplitudes in experts during domain-specific tasks, consistent with reduced cognitive load and optimized working memory updating [18]. Other studies, however, have reported larger P300 amplitudes in experts, particularly in tasks requiring deeper semantic processing or involving complex tactical decision-making [19]. This heterogeneity suggests that the direction of expertise-related P300 modulation may depend on task demands and processing stage, rather than reflecting a universal principle [20,21]. Furthermore, the late negative component (LNC) is associated with long-term cognitive integration, indicating that skilled performers engage in more efficient schema-based processing when recognizing and interpreting complex game scenes [22,23,24]. This study argues that skilled performers do not simply “store more memories” but may achieve a qualitative leap in neural processing through compressed neural encoding—reducing cognitive resource consumption while maintaining high accuracy under certain task conditions. This compressed encoding is essentially an efficient neural representation of structured patterns, enabling skilled performers to accomplish complex pattern recognition tasks at a lower cognitive cost.

In recent years, deep generative models in artificial intelligence, particularly the Transformer architecture [24], have provided a novel computational framework for simulating and testing the aforementioned human cognitive mechanisms. Based on the multi-head self-attention mechanism, the Transformer can process relationships among all positions in an input sequence in parallel and automatically focus on task-relevant features—a conceptual analogy to skilled players’ hierarchical visual sampling strategy achieved through selective attention. Its encoder transforms visual features into high-level semantic vectors and constructs a semantic space through hierarchical encoding—a process highly analogous to skilled players’ semantic mapping of visual patterns into tactical concepts. Moreover, its memory retrieval mechanism enabled by pre-trained weights—similar to human long-term memory schema activation—provides a computational model for understanding skilled players’ compressed neural encoding. Therefore, the Transformer is not a mechanical analogy in this study but rather a theoretical tool that can be used to model, test, and further understand the cognitive mechanisms of skilled players.

Based on the above analysis, this study adopts a multimodal approach (eye-tracking + ERP) to simultaneously capture behavioral output (decision reaction time and accuracy), visual search patterns (eye-tracking metrics), and neural processing (ERP components: P300, LNC), systematically examining the neuro-behavioral coupling mechanisms underlying skilled-player performance in basketball decision-making contexts. The specific research questions are as follows:

1. How do structured patterns influence basketball decision-making? (Input layer: behavioral performance).
2. How do skilled basketball players optimize their visual search strategies through selective attention? (Hypothesis 1: Skilled players adopt a “global-first” hierarchical visual sampling strategy, characterized by fewer first fixations and strategic fixation distribution).
3. How do skilled basketball players achieve behavioral decision advantages through semantic mapping? (Hypothesis 2: Skilled players’ decision advantage primarily stems from enhanced perceptual encoding efficiency rather than mere response selection automation, to be tested through behavioral metrics).
4. How do skilled basketball players optimize neural resources through memory integration? (Hypothesis 3: Skilled players employ compressed neural encoding, manifested as reduced P300 amplitude and modulated LNC, reflecting decreased cognitive load and enhanced schema-based processing efficiency).

Ultimately, within an interdisciplinary framework integrating cognitive psychology, neuroscience, and artificial intelligence, this study will address the two fundamental questions posed at the outset: Why can skilled players process structured patterns more efficiently? How do humans learn to become skilled players?

## 2. Materials and Methods

### 2.1. Experimental Design

A 2 (patterns: structured, unstructured) × 2 (skill: skilled, less-skilled) mixed experimental design was adopted. The between-group independent variable was skill, the within-group independent variable was patterns, and the dependent variables were decision accuracy rate, decision reaction time, eye movement indicators (average pupil diameter, fixation counts, fixation duration), and brainwave indicators (P300 component, LNC).

### 2.2. Participants

The skilled participants included 50 basketball players from China University Basketball Association (CUBA) teams: 46 first-grade national athletes and 4 second-grade national athletes; 35 males (70%) and 15 females (30%); ages ranging from 18 to 29 (20.24 ± 2.10) years old; and training years ranging from 4 to 13 (7.54 ± 2.25) years. The less-skilled participants consisted of 51 basketball enthusiasts who lacked any formal basketball sports ranking, comprehended the basic rules of a basketball match and had not undergone systematic or regular basketball training. There were 21 males (42%) and 30 females (58%), with ages ranging from 18 to 30 (22.92 ± 3.21) years old, and a weekly basketball exercise duration of 2.28 h. The two skill groups differed in sex composition (skilled: 35 males, 15 females; less-skilled: 21 males, 30 females). A chi-square test indicated that this difference was significant, χ^2^(1) = 8.49, *p* = 0.004, φ = 0.29. An independent-samples t-test (equal variances not assumed, because Levene’s test was significant, *F* = 12.67, *p* = 0.001) indicated that the age difference was also significant, *t*(86.25) = 4.98, *p* < 0.001, Cohen’s d = 0.99, with less-skilled participants (22.92 ± 3.21 years) being older than skilled participants (20.24 ± 2.10 years). To examine whether these differences contributed to the reported group effects, we conducted sensitivity analyses with age and sex entered as covariates (see Section 3.4).

Behavioral decision accuracy data were valid for 89 participants (skilled: n = 44; less-skilled: n = 45), decision time data for 89 (skilled: *n* = 44; less-skilled: *n* = 45), eye-tracking data for 90 (skilled: *n* = 45; less-skilled: *n* = 45), and EEG data for 75 (skilled: *n* = 36; less-skilled: *n* = 39). The distribution of exclusions across groups is reported in Table 1.

Participants were excluded if they did not complete the full experimental session, if eye-tracking calibration failed or gaze samples were lost for more than 20% of the trial duration, if EEG data contained excessive artifacts or fewer than 20 accepted trials per condition after preprocessing, or if technical failures occurred during recording. Across participants, the mean number of accepted EEG trials was 62.4 ± 12.7 for structured and 60.8 ± 13.5 for unstructured conditions. All included participants had at least 20 accepted trials per condition after preprocessing. Attrition did not differ significantly between groups for any analysis (accuracy: χ^2^(1) = 0.001, *p* > 0.05; decision time: χ^2^(1) = 0.001, *p* > 0.05; eye-tracking: χ^2^(1) = 0.081, *p* > 0.05; EEG: χ^2^(1) = 0.264, *p* > 0.05), suggesting that differential dropout did not substantially bias the skill comparisons.

All participants were right-handed with normal or corrected-to-normal vision. In addition, the participants gave their written informed consent following the Declaration of Helsinki. The study was approved by the ethics committee, and participants who completed the study were compensated for their time.

### 2.3. Materials

Pattern images were selected from 2015–2016 NBA basketball game videos. Information regarding player positions and spatial configurations was obtained from the NBA official website and converted into static point-light displays using Python 3.8. Each display represented a single frozen moment of a half-court offensive–defensive configuration. No movement trajectories or body-orientation cues were available in the final stimuli; therefore, all interpretations in this study refer to spatial-positional information only. A total of 180 structured-pattern static point-light displays were selected, equally divided among passing, breakthrough, and shooting scenarios. Similarly, 180 unstructured-pattern static point-light displays were selected, also equally divided among the same three scenarios.

During the material evaluation process, correct responses were defined as the tactically optimal action identified by the skilled-player panel, rather than the action actually performed in the original game. Three skilled basketball players were invited to rate the degree of structure of the 360 patterned static point-light displays on a scale from 1 to 10 (1 = unstructured, 10 = structured), as well as the difficulty level on a scale from 1 to 7. The skilled-player panel consisted of a national-level coach (aged 40) with 7 years of coaching experience, an international referee (aged 42) with 20 years of refereeing experience, and a national-level athlete (aged 22) with 8 years of training experience.

**Figure 1:**
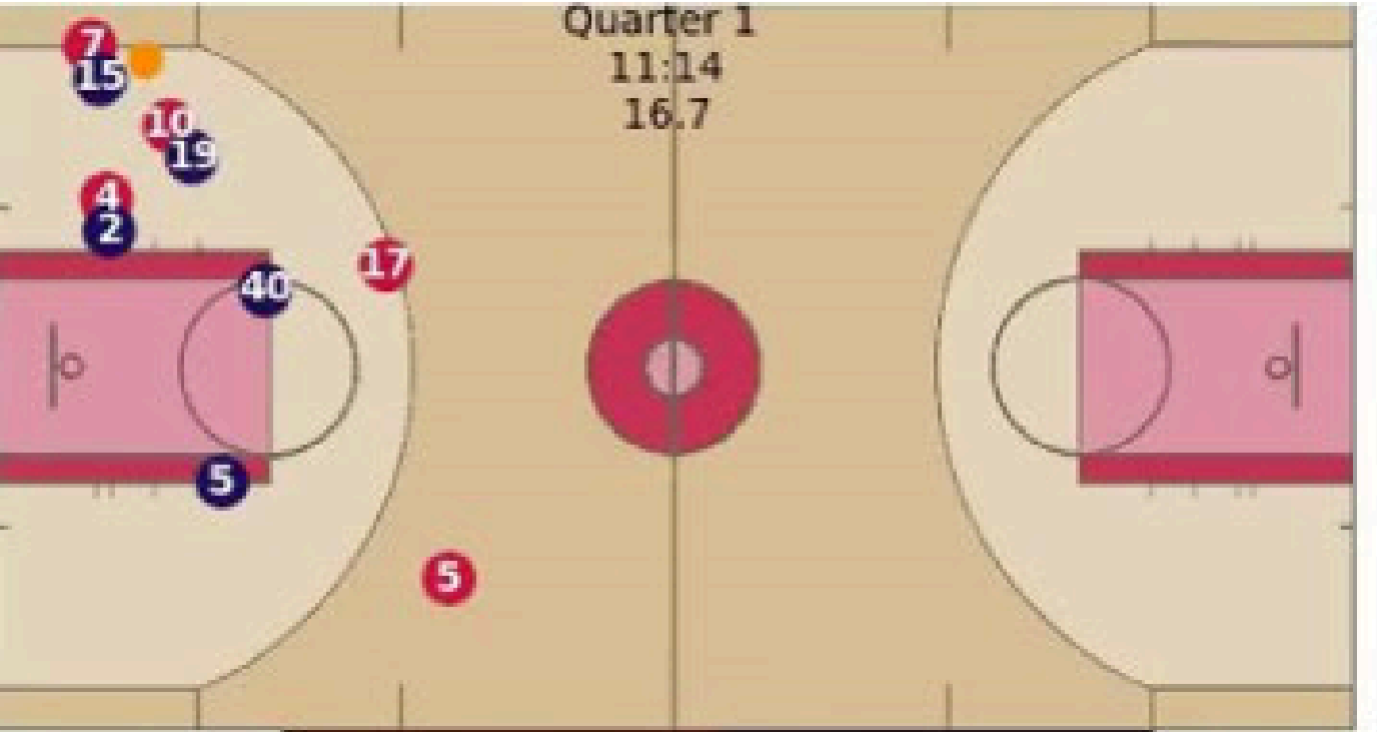
Structured pattern image. Red and blue dots represent the two opposing teams; the orange dot represents the ball. Numbers indicate individual player positions within the half-court configuration.

**Figure 2:**
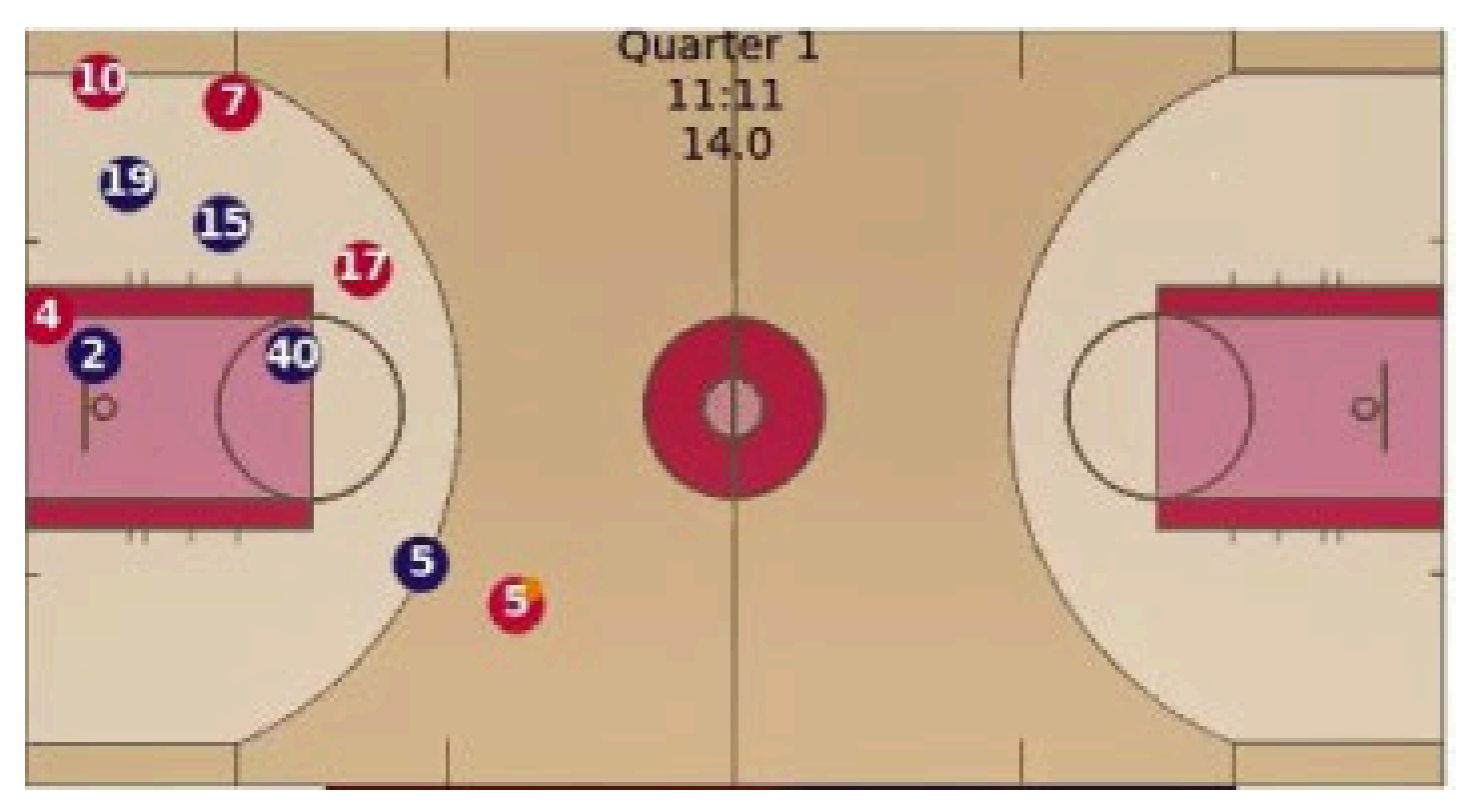
Unstructured pattern image. Red and blue dots represent the two opposing teams; the orange dot represents the ball. Numbers indicate individual player positions after randomization.

Only images with consistent evaluations across all three skilled players were included in the study, based on the following criteria: structured images (score ≥ 7), unstructured images (score ≤ 3), and medium-difficulty images (score between 3 and 6). Images that did not receive consistent ratings were excluded. Following this screening process, a total of 180 images were retained, with 90 images each for structured and unstructured patterns. Examples are shown in Figure 1 and Figure 2.

### 2.4. Experiment Procedures

**Figure 3:**
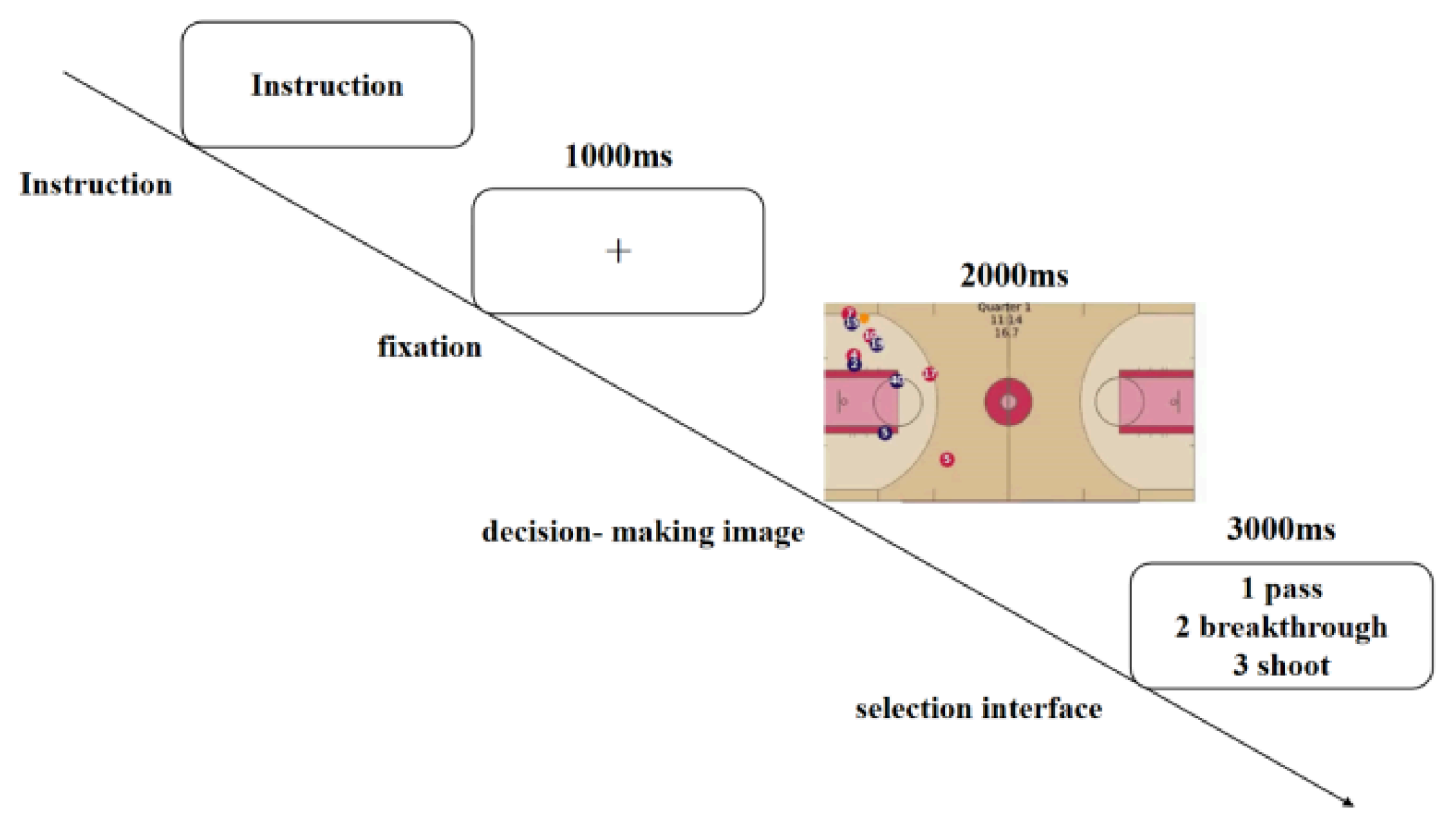
Decision-making task flowchart.

1. Upon arriving at the laboratory, participants filled out an informed consent form.
2. The EEG system was calibrated, and a nine-point eye-tracking calibration was performed.
3. Participants performed the decision-making task (see Figure 3). The experimenter first explained the experimental procedure. A fixation cross appeared on the screen, followed by a 2000 ms static point-light image of a basketball game. Participants assumed the role of the ball handler and made quick and accurate decisions to pass, breakthrough, or shoot based on the image content by pressing the corresponding keys (1, 2, 3) on the keyboard. Reaction time was measured from the offset of the 2000 ms stimulus display, that is, from the onset of the response interface. Participants had to respond within 3000 ms after the onset of the response interface. If no response was made within this window, the trial was terminated and the next trial began. After three practice trials, the formal experiment began. The formal experiment consisted of 180 trials, with 90 structured pattern images and 90 unstructured pattern images, presented randomly in groups without breaks. The decision-making task lasted approximately 15 min.
4. The experimenter explained the purpose of the experiment to the participants and gave them gifts as a token of appreciation.

### 2.5. Research Instruments

**Figure 4:**
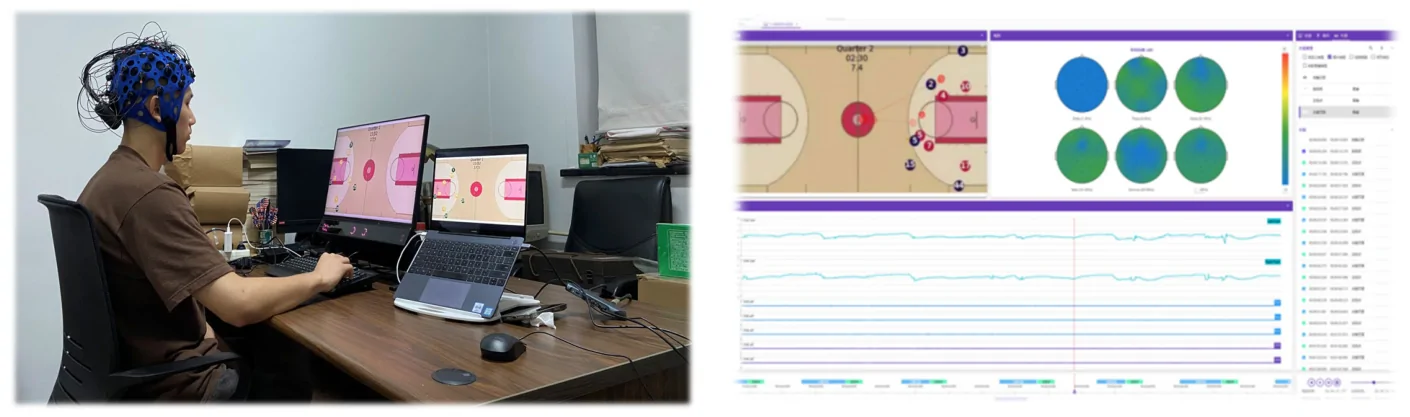
Experimental setup and multimodal data acquisition. The photograph shows a participant performing the decision-making task. Key instrumentation is labeled: (1) 32-channel EEG cap (Bitbrain); (2) Tobii T60XL eye tracker mounted below the display; (3) 24-inch display presenting the static point-light stimuli; (4) response keyboard used for the pass/breakthrough/shoot decision; and (5) ErgoLAB Human–Computer Environment Synchronization Platform V 3.0, which synchronized EEG, eye-tracking, and behavioral data.

The ErgoLAB Human–Computer Environment Synchronization Platform V 3.0 was used for the synchronous collection of multimodal eye movement, EEG, and behavioral data (refer to Figure 4). Eye movements were recorded using a Tobii T60XL eye tracker (Tobii Technology, Stockholm, Sweden), which has a 24-inch widescreen display with a sampling frequency of 60 Hz and a screen resolution of 1920 × 1600 pixels. Participants sat 65 cm from the display in a dimly lit room.

Eye-tracking data processing: fixations were identified using the Tobii I-VT (Velocity-Threshold) fixation filter with a velocity threshold of 30°/s and a minimum fixation duration of 60 ms. For analysis, only the 2000 ms stimulus presentation window was included; the fixation cross and the response interface were excluded. Therefore, total fixation duration values were constrained to a maximum of 2000 ms per trial.

Calibration: A nine-point calibration procedure was performed before the experiment. Calibration was accepted if the average deviation was below 1° of visual angle; if this criterion was not met, calibration was repeated, up to a maximum of five attempts.

Areas of interest (AOIs): Four AOIs were defined for each stimulus: teammate, opponent, ball, and open space. AOIs were manually delineated on the static point-light displays, and the same AOI template was applied to all structured and unstructured images within the same scenario category. The AOIs differed substantially in size, with open space occupying a much larger region than the ball; raw fixation counts and durations are therefore not directly comparable across AOIs.

Pupil diameter: Pupil diameter was recorded continuously throughout each trial. For analysis, pupil data were baseline-corrected using the mean pupil diameter during the 200 ms pre-stimulus interval. Blinks were handled by excluding the affected segments from analysis, and trials with excessive blink artifacts were excluded. Stimulus luminance was kept constant across all trials and conditions by fixing the screen brightness.

Continuous EEG data were recorded using a 32-channel wearable cap (Bitbrain) with earlobes as the online reference. The sampling rate was 512 Hz. Data were preprocessed offline using EEGLAB 19.0 running on MATLAB [R2020b].

Data cleaning and filtering: Gross artifacts (e.g., flat or excessively noisy channels) were manually identified and rejected. To preserve the slow cortical potentials critical for the late negative component (LNC) [22,25], a zero-phase-shift bandpass filter of 0.1–30 Hz was applied. The lower cutoff frequency was set to 0.1 Hz (rather than 1 Hz) to avoid attenuating the slow drifts that constitute the LNC amplitude.

Artifact removal: Independent component analysis (ICA) was performed to identify and remove ocular artifacts (blinks and saccades) and muscle artifacts. Artifactual components were identified based on scalp topography, time course, and power spectrum: components with frontal, high-amplitude slow deflections were classified as ocular artifacts, and components with high-frequency, broad or peripheral scalp distributions were classified as muscle artifacts. Two trained researchers independently inspected the components, and disagreements were resolved by consensus. Following ICA, channels with residual high-amplitude noise (±100 μV) were interpolated using spherical spline interpolation based on neighboring good channels.

Epoching and baseline correction: Epochs were extracted from −200 ms to +1800 ms relative to stimulus onset [22,25]. Epochs containing artifacts exceeding ±80 μV were automatically rejected. Baseline correction was applied using the mean voltage of the −200 ms to 0 ms pre-stimulus interval.

ERP averaging: ERPs were averaged separately for each experimental condition (skill: skilled vs. less-skilled; pattern: structured vs. unstructured) to obtain grand-average waveforms.

Amplitude measures: P300 amplitude was defined as the maximum positive peak within the 280–480 ms window. LNC amplitude was defined as the maximum negative peak within the 600–750 ms window; therefore, larger LNC amplitudes correspond to more negative values.

Electrode selection: Based on previous literature and the observed topographical distribution, Pz was selected a priori as the electrode site of interest for both P300 and LNC analyses.

## 3. Results

### 3.1. Behavior Indicators

#### 3.1.1. Decision Accuracy

**Figure 5:**
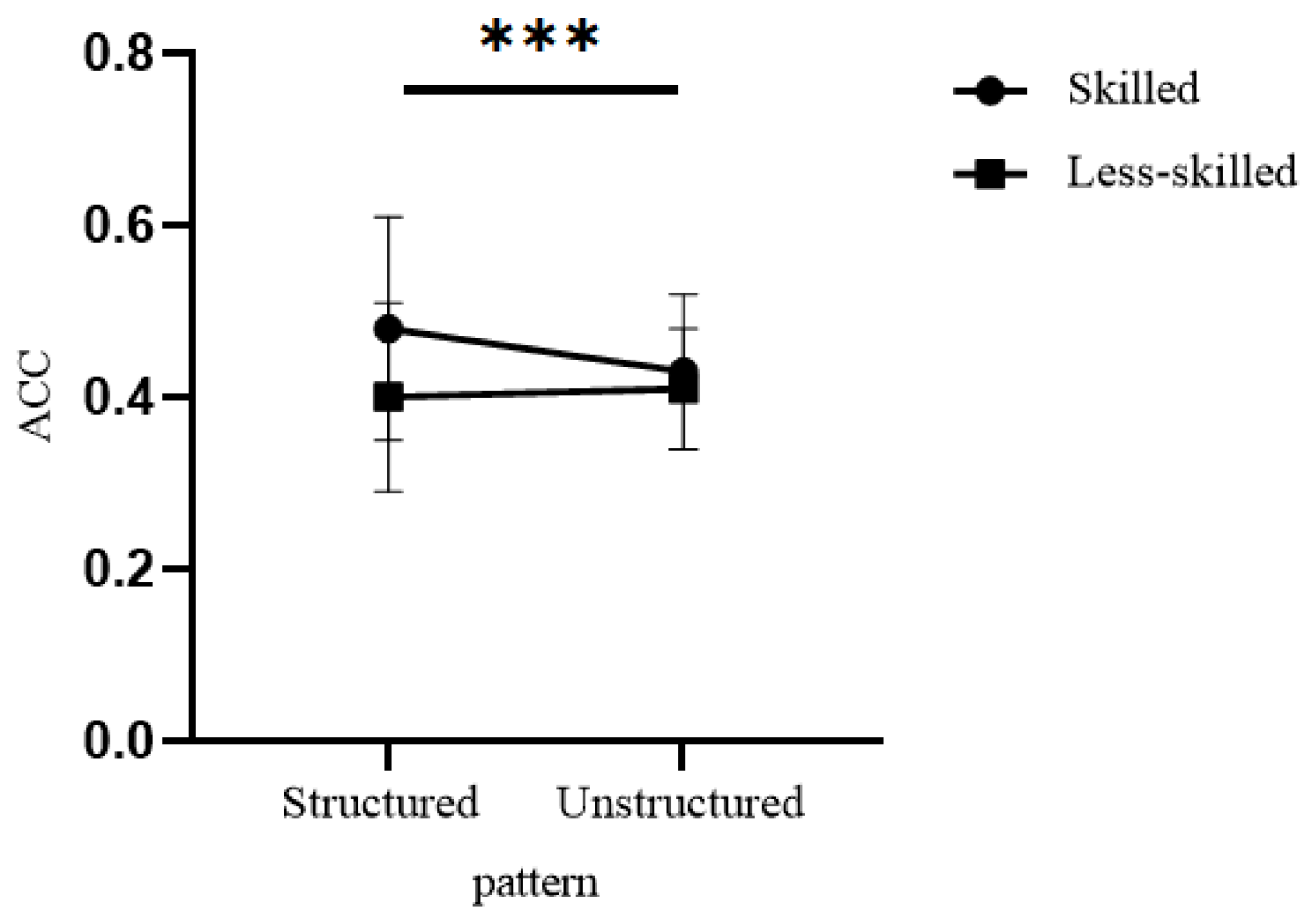
The skill × pattern interaction for decision accuracy. Error bars represent standard errors. *** *p* < 0.001.

A repeated-measure ANOVA revealed a significant difference in decision accuracy rate between skilled and less-skilled participants, *F*_(1, 87)_ = 5.96, *p* = 0.017, η_p_^2^ = 0.06. Skilled participants (0.46 ± 0.11) were more accurate than less-skilled participants (0.41 ± 0.09). There was a main effect for pattern, *F*_(1, 87)_ = 4.79, *p* = 0.031, η_p_^2^ = 0.05. The structured pattern (0.44 ± 0.12) was more accurate than the unstructured pattern (0.42 ± 0.08). The skill × pattern interaction was significant, *F*_(1, 87)_ = 6.53, *p* = 0.012, η_p_^2^ = 0.07. Less-skilled participants did not differ between structured (0.40 ± 0.11) and unstructured patterns (0.41 ± 0.07 *p* = 0.795, 95% CI [−0.03, 0.34]), whereas skilled participants showed higher accuracy in structured (0.48 ± 0.13) than unstructured patterns (0.43 ± 0.09, *p* = 0.001, 95% CI [0.02, 0.08]) (see Figure 5). Under unstructured conditions, the two groups did not differ in decision accuracy, *p* = 0.286, 95% CI [−0.054, 0.016]. Under structured conditions, skilled participants showed significantly higher accuracy than less-skilled participants, *p* = 0.005, 95% CI [0.023, 0.124]. See Table 2 for details.

#### 3.1.2. Decision Time

**Figure 6:**
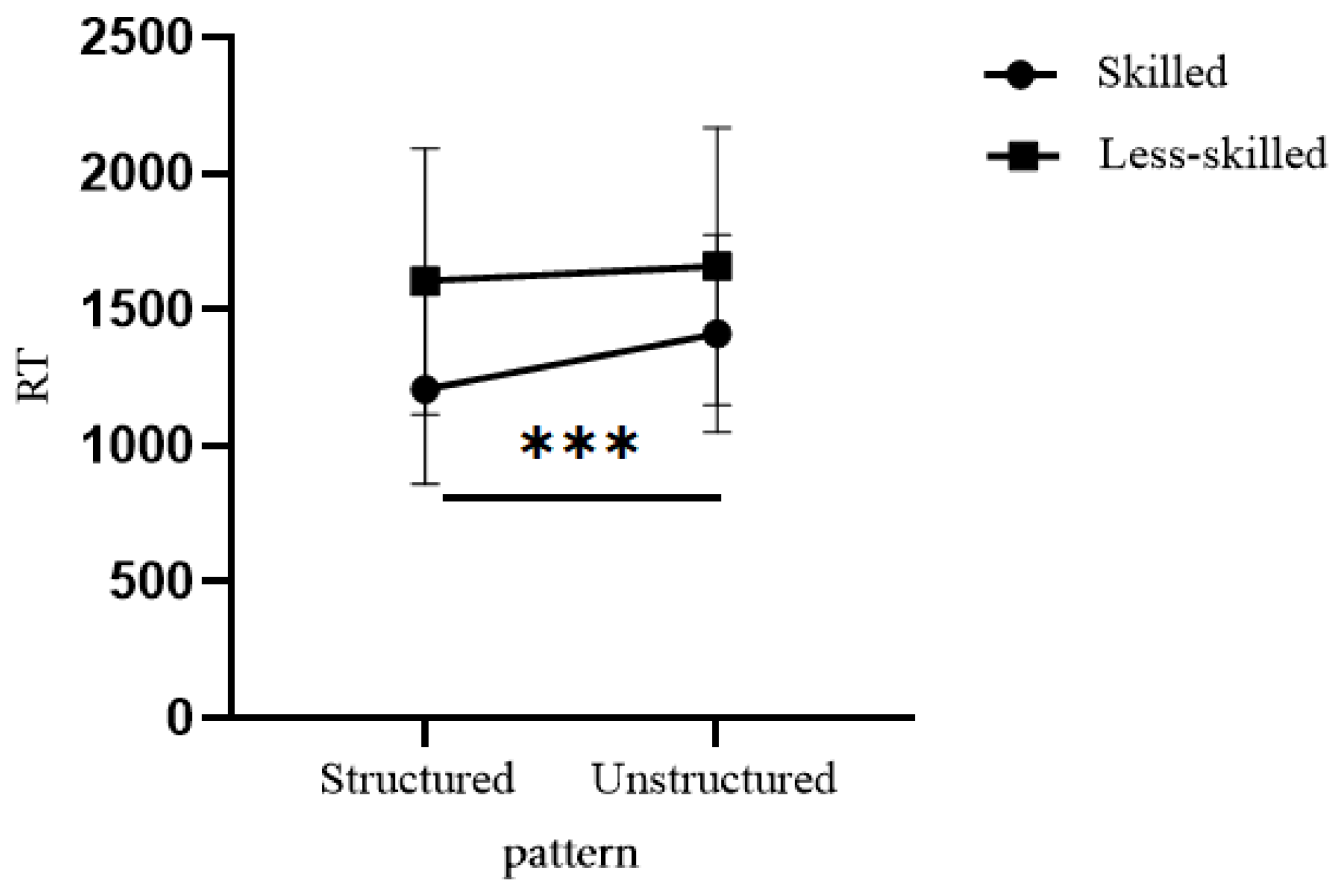
The skill × pattern interaction for decision time. Error bars represent standard errors. *** *p* < 0.001.

A repeated-measure ANOVA revealed a significant difference in decision time between skilled and less-skilled participants, *F*_(1, 87)_ = 14.83, *p* < 0.001, η_p_^2^ = 0.15. Skilled participants (1309.24 ± 59.57 ms) were quicker than less-skilled participants (1631.84 ± 58.91 ms). There was a main effect for pattern, *F*_(1, 87)_ = 11.67, *p* = 0001, η_p_^2^ = 0.12. The structured pattern (1405.59 ± 45.00 ms) was quicker than the unstructured pattern (1535.49 ± 46.98 ms). The skill × pattern interaction was significant, *F*_(1, 87)_ = 3.97, *p* = 0.05, η_p_^2^ = 0.04. Less-skilled participants did not differ between structured (1604.74 ± 488.28 ms) and unstructured patterns (1658.94 ± 509.61 ms, *p* = 0.313, 95% CI [−52.06, 160.46]), whereas skilled participants were quicker in structured (1206.44 ± 347.21 ms) than unstructured patterns (1412.03 ± 362.87 ms, *p* < 0.001, 95% CI [98.13, 313.05])(see Figure 6). Simple effect analyses showed that skilled participants responded significantly faster than less-skilled participants under both unstructured conditions (*p* = 0.010, 95% CI [0.060, 0.434]) and structured conditions (*p* < 0.001, 95% CI [0.208, 0.571]). The group difference was larger under structured than under unstructured conditions. See Table 3 for details.

### 3.2. Point-of-Gaze

#### 3.2.1. Average Pupil Diameter

**Figure 7:**
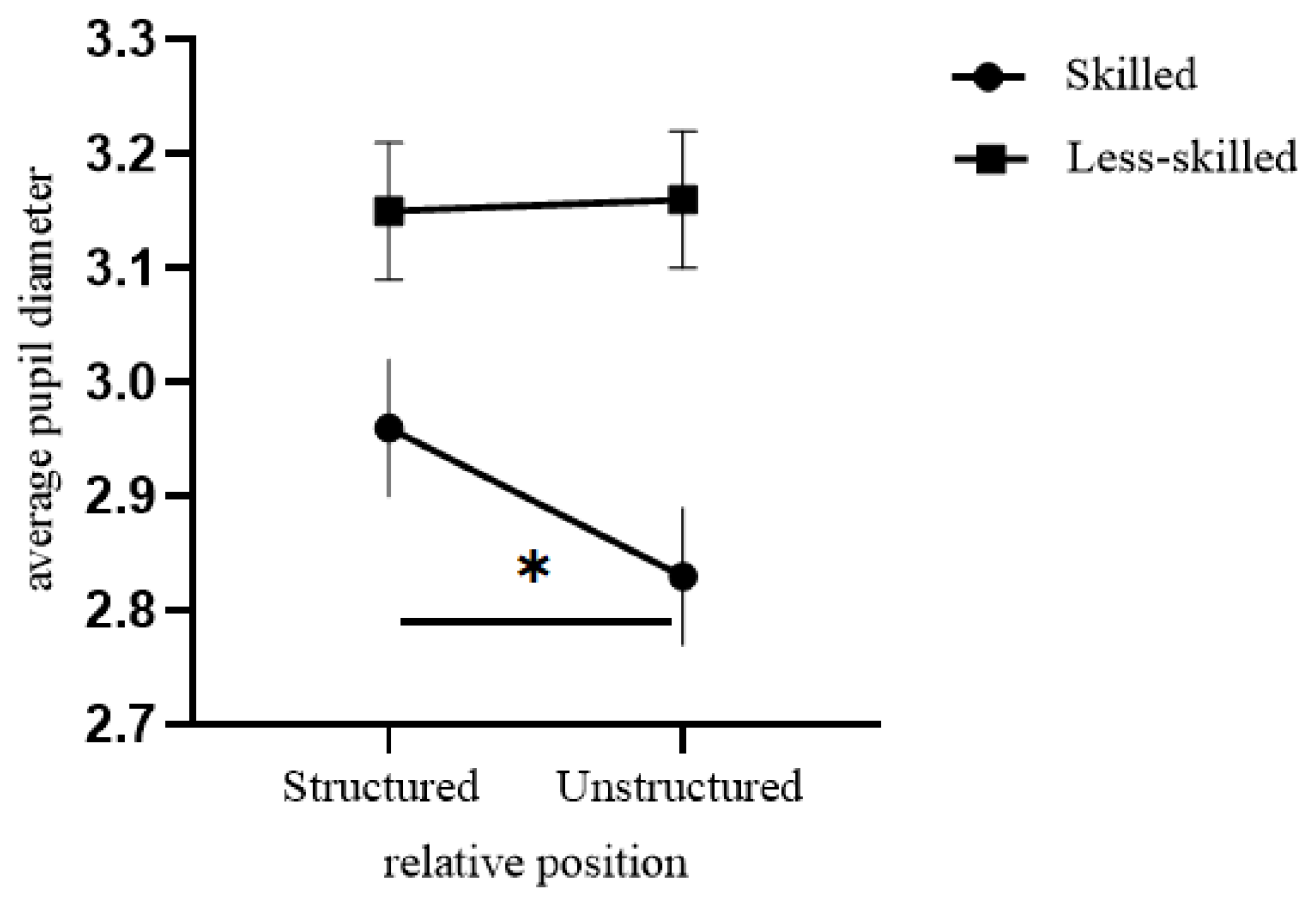
The skill × pattern interaction for average pupil diameter. Error bars represent standard errors. * *p* < 0.05.

In assessing the impact of skill and pattern on visual search efficiency in basketball decision-making, a repeated-measures ANOVA uncovered a noteworthy contrast in decision average pupil diameter between skilled and less-skilled participants, *F*_(1, 88)_ = 18.85, *p* < 0.001, η_p_^2^ = 0.18, with skilled participants (2.89 ± 0.05mm) showing a lower value than less-skilled participants (3.02 ± 0.05mm). Moreover, a main effect for pattern was observed, *F*_(1, 88)_ = 5.10, *p* = 0.027, η_p_^2^ = 0.06, with that for the structured pattern (3.03 ± 0.04 mm) lower than that for the unstructured pattern (3.06 ± 0.04 mm). Particularly significant was the skill × pattern interaction, *F*_(1, 88)_ = 5.09, *p* = 0.03, η_p_^2^ = 0.06. Less-skilled participants did not differ between structured (3.17 ± 0.45 mm) and unstructured patterns (3.23 ± 0.46 mm, *p* = 0.282, 95% CI [−0.19, 0.06])(see Figure 7). In contrast, skilled participants showed larger pupil diameter under structured (2.96 ± 0.28 mm) than unstructured conditions (2.83 ± 0.36 mm, *p* = 0.038, 95% CI [0.01, 0.25]). Skilled participants had significantly smaller pupil diameters than less-skilled participants under both unstructured conditions (*p* = 0.009, 95% CI [−0.365, −0.053]) and structured conditions (*p* < 0.001, 95% CI [−0.577, −0.231]). The group difference was larger under structured than under unstructured conditions. See Table 4 for details.

#### 3.2.2. Total Fixation Counts

To measure the impact of skill and pattern on visual search efficiency in basketball decision-making, a repeated-measures ANOVA was conducted with total fixation duration as the dependent variable. The results showed a significant main effect for skill and pattern. The total fixation counts for the structured pattern (6.53 ± 1.17) were fewer than those for the unstructured pattern (6.83 ± 0.77) (*F*_(1, 88)_ = 6.18, *p* = 0.015, η_p_^2^ = 0.07). The total fixation counts for the skilled participants (6.31 ± 1.02) were fewer than those for the less-skilled participants (7.15 ± 0.84) (*F*_(1, 88)_ = 23.90, *p* < 0.001, η_p_^2^ = 0.21). There was no significant the skill × pattern interaction (*p*s ≥ 0.33).

In each trial image, four areas of interest were examined: teammate, opponent, ball, and open space. A significant main effect for skill was observed across opponent (*F*_(1, 88)_ = 5.79, *p* = 0.012, η_p_^2^ = 0.06) and open space (*F*_(1, 88)_ = 19.79, *p* < 0.001, η_p_^2^ = 0.18). Skilled participants exhibited lower attention allocation than less-skilled participants towards opponent and open space. There was a main effect for pattern on teammates (*F*_(1, 88)_ = 8.91, *p* = 0.004, η_p_^2^ = 0.09) and open spaces (*F*_(1, 88)_ = 10.05, *p* = 0.002, η_p_^2^ = 0.10) for the structured pattern less than the unstructured pattern. For further details, please refer to Table 5.

It should be noted that the four AOIs differed substantially in size, with open space occupying a much larger region than the ball. Raw fixation counts are therefore not directly comparable across AOIs, and the between-AOI comparisons should be interpreted as reflecting relative attentional distribution rather than absolute attentional priority per unit area.

#### 3.2.3. Total Fixation Duration

To measure the impact of skill and pattern on visual search efficiency in basketball decision-making, we performed a repeated-measures analysis of variance with total fixation duration as the dependent variable. Results showed that the main effect of skill was not significant, although skilled participants (1510.62 ± 972.83 ms) tended to show shorter total fixation duration than less-skilled participants (1696.75 ± 448.61 ms), *F*_(1, 88)_ = 3.18, *p* = 0.078, η_p_^2^ = 0.035. Additionally, a significant main effect of pattern was found, with the total fixation duration for structured patterns (1450.63 ± 315.73 ms) shorter than that for unstructured patterns (1756.75 ± 1009.01 ms) (*F*_(1, 88)_ = 6.80, *p* = 0.011, η_p_^2^ = 0.07). However, there was no significant two-way interaction between pattern and skill (*p*s ≥ 0.51).

In each trial image, four areas of interest were examined: teammate, opponent, ball, and open space. A significant main effect for skill was observed across opponent (*F*_(1, 88)_ = 9.17, *p* = 0.003, η_p_^2^ = 0.09) and ball (*F*_(1, 88)_ = 6.00, *p* = 0.016, η_p_^2^ = 0.06). Skilled participants exhibited lower fixation duration than less-skilled participants towards opponent. Conversely, less-skilled participants showed less fixation duration towards ball compared to skilled participants. There was a main effect for pattern on teammate (*F*_(1, 88)_ = 13.15, *p* < 0.001, η_p_^2^ = 0.13), opponent (*F*_(1, 88)_ = 9.17, *p* = 0.003, η_p_^2^ = 0.09), ball (*F*_(1, 88)_ = 6.00, *p* = 0.016, η_p_^2^ = 0.06), and open space (*F*_(1, 88)_ = 5.51, *p* = 0.021, η_p_^2^ = 0.06), for the structured pattern less than the unstructured pattern towards teammate, opponent and open space. For further details, please refer to Table 6.

### 3.3. EEG Metrics

ERP components: The key components examined were P300 and the late negative component (LNC), based on previous research [26,27,28]. Peak amplitudes were identified as the maximum positive peak within the 280–480 ms window for P300 [29,30,31], and the maximum negative peak within the 600–750 ms window for LNC [22,25]. Based on previous literature and the observed topographical distribution, Pz was selected a priori as the electrode site of interest for both P300 and LNC analyses [25,26,27,28,29,30]. P300 and LNC amplitudes were therefore quantified at Pz and submitted to statistical analysis. A 2 (pattern: structured vs. unstructured) × 2 (skill: skilled vs. less-skilled) repeated-measures ANOVA was conducted separately for each component and electrode site. All statistical tests were performed with a significance threshold of α = 0.05.

**Figure 8:**
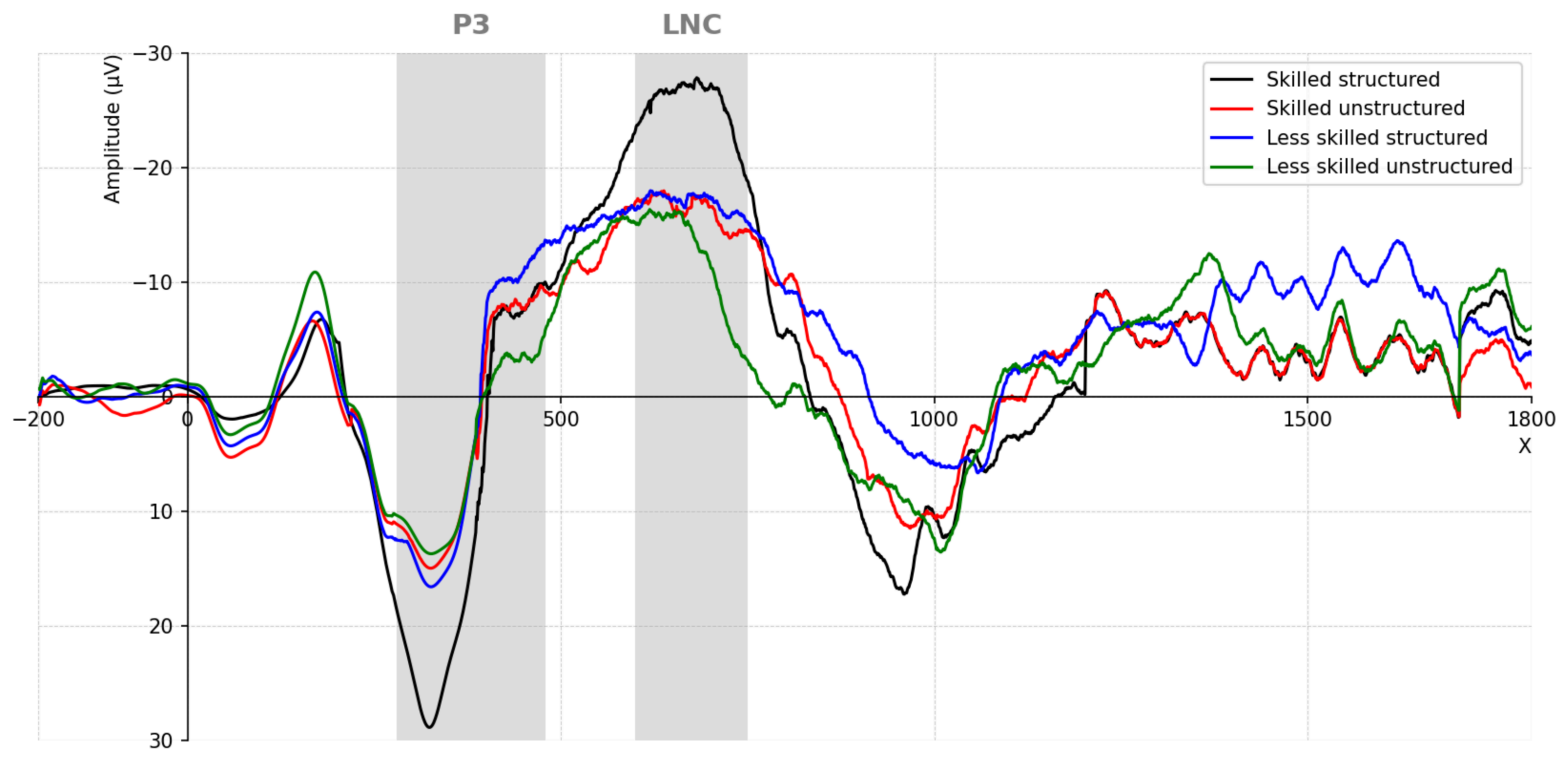
. ERP waveforms at Pz. The waveform displays the analyzed epoch from −200 ms to 1800 ms. Shaded regions indicate the P300 (280–480 ms) and LNC (600–750 ms) analysis windows.

Using the maximum positive peak in the 280–480 ms time window at the pz electrode point (the peak amplitude of P300) as the dependent variable, a 2 (patterns) × 2 (skill) repeated-measures analysis of variance was conducted. The results showed a significant main effect for patterns, with the amplitude under structured patterns (14.57 ± 3.66 μV) being higher than that under unstructured patterns (13.35 ± 3.51 μV) (*F*_(1, 73)_ = 5.069, *p* = 0.027, η_p_^2^ = 0.065). There was a significant result for skill, with the amplitude under skill (14.69 ± 3.45 μV) being higher than that under less skill (13.29 ± 3.47 μV) (*F*_(1, 73)_ = 6.747, *p* = 0.011, η_p_^2^ = 0.085). The pattern × skill interaction was significant, *F*_(1, 73)_ = 10.135, *p* = 0.002, η_p_^2^ = 0.122. Under structured patterns, the skilled participants’ amplitude (16.25 ± 3.12 μV) was higher than that of the less-skilled participants (13.02 ± 3.46 μV) (*p* < 0.001 95% CI [1.473, 4.780]); under unstructured patterns, there was no significant difference between the skilled participants (13.13 ± 3.57 μV) and less-skilled participants (13.56 ± 3.48 μV) (*p* = 0.503, 95% CI [−1.052, 2.125]). See Figure 8 for details.

Using the maximum negative peak in the 600–750 ms time window at the Pz electrode as the peak amplitude of the LNC, a 2 (patterns) × 2 (skill) repeated-measures analysis of variance was conducted. The results showed a significant main effect for patterns, with the amplitude under structured patterns (−22.99 ± 2.38 μV) being higher than that under unstructured patterns (−21.73 ± 2.65 μV) (*F*_(1, 73)_ = 10.243, *p* = 0.002, η_p_^2^ = 0.123). There was a significant result for skill, with the amplitude under skill (−22.92 ± 2.91 μV) being higher than that under less skill (−21.85 ± 1.76 μV) (*F*_(1, 73)_ = 7.846, *p* = 0.007, η_p_^2^ = 0.097). The pattern × skill interaction was significant, *F*_(1, 73)_ = 7.076, *p* = 0.01, η_p_^2^ = 0.088. Under structured patterns, skilled participants (−24.11 ± 2.35 μV) showed more negative LNC amplitudes than less-skilled participants (−21.96 ± 1.91 μV) (*p* < 0.001 95% CI [1.212, 3.545]); under unstructured patterns, there was no significant difference between the skilled participants (−21.73 ± 3.47 μV) and less-skilled participants (−21.74 ± 1.61 μV) (*p* = 0.698, 95% CI [−0.901, 1.340]). See Figure 8 for details.

### 3.4. Sensitivity Analyses Controlling for Age and Sex

Because the skilled and less-skilled groups differed significantly in age and sex distribution, we conducted sensitivity analyses using ANCOVA with age and sex as covariates for the main behavioral, eye-tracking, and ERP outcomes.

For decision accuracy, the main effect of skill remained significant after controlling for age and sex, *F*_(1, 172)_ = 4.59, *p* = 0.034, η_p_^2^ = 0.026, whereas the main effect of pattern was no longer significant, *F*_(1, 172)_ = 2.32, *p* = 0.129, η_p_^2^ = 0.013, and the skill × pattern interaction became marginally significant, *F*_(1, 172)_ = 3.17, *p* = 0.077, η_p_^2^ = 0.018.

For pupil diameter, the main effect of skill remained significant, *F*_(1, 174)_ = 21.44, *p* < 0.001, η_p_^2^ = 0.110, whereas the main effect of pattern was no longer significant, *F*_(1, 174)_ = 0.30, *p* = 0.587, η_p_^2^ = 0.002, and the skill × pattern interaction became marginally significant, *F*_(1, 174)_ = 2.87, *p* = 0.092, η_p_^2^ = 0.016. Sex was a significant covariate, *F*_(1, 174)_ = 5.97, *p* = 0.016, η_p_^2^ = 0.033.

For total fixation counts, the main effect of skill remained significant, *F*_(1, 174)_ = 25.31, *p* < 0.001, η_p_^2^ = 0.127, and the main effect of pattern remained significant, *F*_(1, 174)_ = 4.72, *p* = 0.031, η_p_^2^ = 0.026. The skill × pattern interaction was not significant, *F*_(1, 174)_ = 0.72, *p* = 0.398, η_p_^2^ = 0.004.

For total fixation duration, the main effect of pattern remained significant, *F*_(1, 174)_ = 7.63, *p* = 0.006, η_p_^2^ = 0.042, whereas the main effect of skill was not significant, *F*_(1, 174)_ = 2.13, *p* = 0.146, η_p_^2^ = 0.012.

For P300 amplitude, the main effect of skill remained significant, *F*_(1, 144)_ = 5.41, *p* = 0.021, η_p_^2^ = 0.036, the main effect of pattern remained significant, *F*_(1, 144)_ = 5.32, *p* = 0.023, η_p_^2^ = 0.036, and the skill × pattern interaction remained significant, *F*_(1, 144)_ = 10.64, *p* = 0.001, η_p_^2^ = 0.069.

For LNC amplitude, the main effect of skill remained significant, *F*_(1, 144)_ = 7.98, *p* = 0.005, η_p_^2^ = 0.053, the main effect of pattern remained significant, *F*_(1, 144)_ = 10.74, *p* = 0.001, η_p_^2^ = 0.069, and the skill × pattern interaction remained significant, *F*_(1, 144)_ = 7.49, *p* = 0.007, η_p_^2^ = 0.049.

Neither age nor sex was a significant predictor in most models. Overall, the ERP findings were robust to demographic covariates, whereas the pattern effect and the interaction for decision accuracy and pupil diameter should be interpreted with caution.

### 3.5. Distributional Checks and Non-Parametric Sensitivity Analyses

Kolmogorov–Smirnov and Shapiro–Wilk tests indicated that P300 amplitude did not deviate significantly from normality in either group, whereas LNC amplitude, accuracy, reaction time, and fixation duration deviated from normality in at least one group. Fixation counts deviated from normality in the skilled group only. Given the balanced sample sizes across conditions, repeated-measures ANOVA was considered appropriate. As a sensitivity analysis, non-parametric Friedman tests were conducted for the pattern effect.

The Friedman tests showed a significant effect of pattern for decision accuracy, χ^2^(1) = 8.909, *p* = 0.003; decision time, χ^2^(1) = 18.888, *p* < 0.001; pupil diameter, χ^2^(1) = 4.444, *p* = 0.035; total fixation counts, χ^2^(1) = 4.444, *p* = 0.035; and total fixation duration, χ^2^(1) = 6.400, *p* = 0.011. For P300 and LNC, the Friedman tests did not reach significance (χ^2^(1) = 3.000, *p* = 0.083 for both). Given the smaller sample size for the ERP analyses (*N* = 75), the non-parametric tests had lower power. Overall, the non-parametric results were consistent with the parametric analyses for the behavioral and eye-tracking measures, and the ERP pattern effects were in the same direction but did not reach significance under the non-parametric test.

## 4. Discussion

This study employed a multimodal approach (behavioral, eye-tracking, and ERP) to systematically investigate the processing characteristics of structured patterns in skilled and less-skilled basketball players during decision-making. The main findings were as follows: (1) At the behavioral level, skilled players exhibited higher decision accuracy and faster reaction times under structured conditions, whereas less-skilled players showed no difference between the two conditions. (2) At the eye-tracking level, skilled players demonstrated a specific gaze pattern—allocating more fixations to opponents and the ball, and fewer fixations to open space—with larger pupil diameters under structured conditions. (3) At the ERP level, skilled players showed significantly larger P300 and LNC amplitudes under structured conditions compared to less-skilled players, with no difference under unstructured conditions. Taken together, the results across the three levels indicate that the decision-making advantage of skilled basketball players was most evident under structured conditions and was accompanied by distinct visual search distributions as well as enhanced P300 and LNC responses. These findings are consistent with a theoretical perspective in which expert advantage may reflect a shift from “pattern recognition” toward “abstract representation.” However, this shift does not appear to be characterized by “neural efficiency (reduced activity),” but rather by processes related to “enhanced neural discriminability and deepened semantic mapping.” It should be emphasized that the present study did not directly measure abstract representation or semantic encoding; therefore, these mechanisms should be regarded as theoretical interpretations requiring future empirical testing, rather than as direct conclusions of this study.

### 4.1. Behavioral Level: Structured Pattern Processing as an Skilled Player-Specific Cognitive Skill

This study found that skilled players exhibited a decision-making advantage (higher accuracy, faster reaction times) only under structured conditions, whereas less-skilled players performed at a consistently low level across both conditions with no difference. This pattern is consistent with classic studies [16,32,33] and extends the understanding that less-skilled players are not “incapable of decision-making” but rather “incapable of utilizing structured patterns for decision-making.” Less-skilled participants showed no difference between structured (0.40) and unstructured (0.41) conditions, suggesting that they may rely on a strategy unrelated to pattern structure, such as focusing solely on the ball handler’s actions. In contrast, skilled players’ decision-making was significantly facilitated under structured conditions (accuracy increased from 0.43 to 0.48 in skilled participants), indicating that they have formed abstract representations of structured patterns and can rapidly activate corresponding tactical knowledge upon perceiving key formation features.

A finding at the behavioral level was that skilled players responded significantly faster under structured (1206 ms) than unstructured (1412 ms) conditions, whereas less-skilled players showed no difference. This 200 ms difference has practical significance in time-pressured basketball decision-making. Combined with the accuracy data, skilled players achieved a speed–accuracy double advantage under structured conditions, contradicting the traditional speed–accuracy trade-off and further supporting the view that skilled players have achieved automated or semi-automated semantic mapping under structured conditions.

### 4.2. Eye-Tracking Level: A Strategic Shift from “Spatial Search” to “Person–Ball Tracking”

Eye-tracking data revealed qualitative differences in visual search strategies between skilled players and less-skilled players. First, in terms of total fixation counts, skilled players under structured conditions (6.09) showed fewer fixations than less-skilled players (6.96), consistent with most research [12], indicating that skilled players acquire equal or more information with fewer fixations. However, the analysis of area-of-interest (AOI) distribution revealed a more nuanced pattern: skilled players showed lower attention allocation than less-skilled players toward open space, whereas less-skilled players showed lower attention allocation than skilled players toward opponents. This pattern suggests that skilled players rely more on opponent-related information, whereas less-skilled players distribute more attention to open space.

This finding can be interpreted from the perspective of “abstract representation” [24,34,35]; less-skilled players adopt a space-based search strategy: they need to actively scan open areas on the court because they lack the ability to infer spatial opportunities from opponent positioning. In other words, less-skilled players “see” discrete spatial locations. Skilled players, in contrast, adopt a person-based prediction strategy: by fixating on opponents’ relative positioning and spatial configuration, they can automatically activate tactical schemas (e.g., “a pick-and-roll is about to occur”) and thereby infer the location of open space without directly fixating on it. This corresponds precisely to the multi-head self-attention mechanism in the Transformer architecture: skilled players can simultaneously focus on multiple sources of information (opponents, ball) and, through their interrelationships (e.g., a defender stepping up), directly generate a representation of the target location (open space) without sequentially moving attention to each spatial location.

The anomalous pattern of pupil diameter deserves special attention. This study found that skilled players exhibited significantly larger pupil diameter under structured (2.96 mm) than unstructured (2.83 mm) conditions. For pupil diameter, the pattern of results differed depending on the comparison. Within the skilled group, pupil diameter was larger under structured than unstructured conditions, suggesting greater task engagement with meaningful tactical configurations. Between groups, however, skilled participants showed smaller pupil diameters than less-skilled participants under both structured and unstructured conditions, with a larger group difference under structured conditions. These two findings are not contradictory: they indicate that structured patterns elicited relatively greater pupillary engagement within the skilled group, while skilled participants overall showed lower pupillary responses than less-skilled participants. Given that pupil diameter is typically considered an indicator of cognitive load or arousal [36], this result appears to contradict the intuition that “structured patterns reduce cognitive load.” We propose two possible explanations:

First, structured patterns elicit higher cognitive engagement in skilled players. Upon recognizing a familiar tactical formation, skilled players do not simply “conserve effort” but instead invest more resources in deep processing—including predicting multiple possible offensive outcomes and retrieving corresponding defensive strategies. That is, structured patterns do not reduce task difficulty but rather increase skilled players’ cognitive engagement with the task. This interpretation is highly consistent with the enhanced P300 amplitude findings.

Second, pupil diameter may reflect interest/arousal rather than pure cognitive load. Structured patterns are “meaningful” to skilled players, eliciting stronger task engagement and motivation, whereas random positioning appears “meaningless” and fails to engage skilled players’ cognitive resources. This interpretation awaits validation by future research using subjective interest ratings or galvanic skin response.

### 4.3. Neural Level: ERP Results and a Theoretical Reconceptualization of “Compressed Neural Encoding”

The most striking—and theoretically tension-filled—finding of this study comes from the ERP data. Both P300 and LNC components showed that skilled players exhibited significantly larger P300 amplitudes and more negative LNC amplitudes under structured conditions compared to less-skilled players, with no difference under unstructured conditions. This finding challenges the “compressed neural encoding” hypothesis proposed in the Introduction on multiple levels.

#### 4.3.1. P300: From “Load Reduction” to “Enhanced Context Updating”

The P300 component is typically associated with working memory updating and context updating [37]. The present finding of larger P300 amplitudes in skilled participants is consistent with a subset of sport-expertise ERP studies that have reported enhanced rather than reduced neural activity in experts during domain-specific tasks [19]. However, it contrasts with other studies supporting the traditional neural efficiency account [18]. This discrepancy may be explained by differences in task characteristics: whereas neural efficiency effects are often observed in tasks that become highly automated with practice, enhanced P300 amplitudes may emerge in tasks that require experts to engage in deeper semantic mapping of complex, structured information—precisely the condition examined in the present study [20,21].

Skilled players’ larger P300 amplitudes may reflect more extensive processing of structured patterns, not reduced cognitive load. When skilled players recognize a familiar tactical formation, they do not merely “see” player positions but activate an entire tactical schema. This schema activation requires rapid matching of current perceptual input with abstract representations stored in long-term memory, along with “semantic updating” of the current situation—that is, determining “this is a high pick-and-roll, prepare for a drive to the right.” At the neural level, this process manifests as enhanced P300 generation because it involves larger-scale neural synchronization (involving the integration of perception, memory, and decision-making across multiple brain regions) rather than reduced activity in a single brain region [38].

In other words, skilled players’ “efficiency” is achieved not by reducing neural activity but by increasing the “information density” of neural activity. For the same P300 generation, skilled players carry the abstract concept of “pick-and-roll,” whereas less-skilled players carry only the raw perception of “a player is moving.” Therefore, skilled players’ larger amplitudes are a neural signature of enhanced representational quality, not inefficiency [39].

This is highly consistent with the Transformer metaphor: at the encoder layer, low-level input features (player coordinates) are mapped to high-level semantic vectors (tactical concepts). This mapping process requires substantial computational resources (higher “neural activity”) in the model, but the quality of its output (semantic vectors) determines the quality of subsequent decisions. Thus, skilled players achieve “high output” through “high consumption” rather than “high output” through “low consumption.”

#### 4.3.2. LNC: Enhanced Long-Term Memory Integration

The LNC is typically associated with schema-based processing and long-term memory integration [22,23]. The significantly more negative LNC amplitudes in skilled players under structured conditions in this study are consistent with the abstract representation account: skilled players’ long-term memory stores richer and more abstract tactical schemas, which are rapidly activated upon recognition of structured patterns and participate in ongoing decision-making processing.

Interestingly, less-skilled players showed no difference in LNC between structured and unstructured conditions, further indicating that less-skilled players have not yet formed automatically activatable tactical schemas. Regarding the source of less-skilled players’ LNC, we speculate that it may reflect general, non-specific late processing (e.g., an “uncertainty” response to random spatial configurations) rather than schema-based integration.

### 4.4. Revisiting the Transformer Metaphor: From “Structural Analogy” to “Conceptual Analogy”

Based on the empirical findings of this study, we refine the computational metaphor of the Transformer architecture for understanding skilled-player cognition as follows:

Multi-head self-attention → hierarchical visual sampling strategy: The “global-first, rapid large-saccade localization” strategy hypothesized in this study, along with skilled players’ allocation of attention simultaneously to “opponents + ball” rather than distributing attention across all areas, is highly consistent with the principle of “parallel computation of all positional interrelationships” in the multi-head self-attention mechanism. Through long-term training, skilled players have learned to compute the interactions between “defender positioning” and “ball handler,” thereby directly deriving the location of “open space” without sequentially scanning each location.

Encoder/semantic mapping → P300 enhancement: The substantial P300 enhancement in skilled players under structured conditions in this study can be understood as an increase in “computational intensity” during the mapping of visual features to semantic vectors at the encoder layer. Semantic mapping is not “compression” but rather an increase in information density—the transformation from “coordinates” to “tactical concepts” requires more complex neural computation, manifesting as larger amplitudes.

Memory integration → LNC enhancement: LNC enhancement corresponds to the retrieval of relevant schemas from pre-trained weights (long-term memory) in the Transformer. This study is consistent with the notion that skilled players more strongly activate long-term memory tactical schemas under structured conditions.

Therefore, we propose a revised theoretical formulation: The conceptual analogy between the Transformer architecture and skilled-player cognition lies in “computational depth” rather than “computational economy.” The skilled-player advantage is not about doing more with less computation, but about performing deeper computations with better representational structures.

### 4.5. Limitations and Future Directions

The “hierarchical visual sampling strategy” not directly tested: The hypothesis proposed in the Introduction of “initial global scan → subsequent local verification” was not directly tested in Section 3. Future research should conduct targeted analyses on first-fixation duration and first-fixation landing position distribution.

Static stimuli: The present study used static point-light displays, which do not contain dynamic motion trajectories or body-orientation cues. Therefore, the findings speak to the processing of spatial-positional configurations rather than dynamic motion processing. Future research should use dynamic stimuli to test whether the same mechanisms extend to moving sequences.

Age and sex differences between groups: The skilled and less-skilled groups differed significantly in sex composition. Because visuospatial attention, pupillometry, and P300 amplitude have been reported to vary with age and sex, we conducted sensitivity analyses controlling for these variables. The ERP findings (P300 and LNC) were robust: the skill main effect, pattern main effect, and skill × pattern interaction all remained significant after controlling for age and sex. The pattern main effect on total fixation counts and total fixation duration was also robust. However, for decision accuracy and pupil diameter, the pattern main effect and the skill × pattern interaction became non-significant or marginally significant after controlling for age and sex, suggesting that these effects may be partly accounted for by demographic differences between groups. Future research should match groups based on age and sex to further isolate skilled-player-related effects.

AOI size differences: The four areas of interest (teammate, opponent, ball, open space) differed substantially in size, with open space occupying a much larger region than the ball. Because raw fixation counts and durations were not normalized by AOI area, the between-AOI comparisons may partly reflect differences in available area rather than attentional prioritization per se. The original data did not allow us to compute fixation density per unit area, because the exact pixel- or degree-based boundaries of each AOI were not retained in a form suitable for area normalization. Therefore, the AOI results should be interpreted as reflecting relative attentional distribution across regions of different sizes, and future research should normalize fixation measures by AOI area to better isolate attentional allocation.

Multiple comparisons:. Multiple ANOVAs were conducted on eye-tracking measures (four AOIs × two dependent variables) and post hoc simple effect tests were conducted following significant interactions. No family-wise error correction was applied across these analyses. Therefore, the AOI-level findings should be interpreted as exploratory, and future research should apply appropriate corrections (e.g., FDR or Bonferroni) to control family-wise error.

Ambiguity in pupil diameter interpretation: This study could not distinguish whether the increased pupil diameter reflected “cognitive load” or “interest/arousal.” Future research could combine subjective ratings (e.g., “how interesting is this scene?”) or galvanic skin response to differentiate between these possibilities.

Boundaries of the Transformer metaphor: This study does not claim that the skilled-player brain is a “biological implementation” of the Transformer, but rather that the two share a common, mappable abstract principle of information processing. Future research could employ representational similarity analysis (RSA) to directly compute the similarity between skilled players’ neural representations of structured patterns and the hidden-layer representations of Transformers, thereby providing stronger evidence.

Lack of transfer tasks: According to the abstract representation hypothesis, skilled players’ representations should exhibit stronger transferability. This study did not include transfer tasks (e.g., transferring from structured patterns to similar but previously unlearned patterns). Future research could incorporate such conditions.

Skilled older adults: The present study compared skilled and less-skilled basketball players within a relatively narrow age range. Future research should include skilled older adults to examine whether the expertise-related advantages observed here—such as enhanced decision accuracy, distinct visual search patterns, and larger P300 amplitudes under structured conditions—are preserved, attenuated, or qualitatively changed with age. This would help clarify whether expertise and aging interact in the processing of structured tactical information, and whether the proposed mechanisms remain stable across the lifespan.

Environmental factors: The present study was conducted in a quiet laboratory setting. In real games, however, athletes perform in noisy environments that can affect arousal, attentional focus, and visual search. Skilled players may be more resistant to auditory distraction, whereas less-skilled players may show greater changes in gaze distribution. Future research should manipulate noise levels to test whether the present findings generalize to more ecologically valid conditions.

## 5. Conclusions

This study examined how basketball expertise relates to the processing of structured game situations using a multimodal approach. Behavioral, eye-tracking, and ERP evidence converged to show that skilled players’ decision-making advantage was specific to structured conditions and was accompanied by distinct visual search patterns and enhanced P300 and LNC responses. These findings are consistent with a theoretical perspective in which expertise involves a shift from surface-level pattern recognition toward more abstract representation of tactical configurations, and in which experts’ efficiency is reflected not by reduced neural activity but by deeper processing of meaningful information. However, abstract representation, neural information density, and neural discriminability were not directly measured in the present study. The Transformer architecture is therefore offered as a conceptual analogy that may guide future hypothesis-driven research. These findings provide a basis for structured, situation-based training in sport decision-making and highlight the need for future studies using transfer tasks, representational similarity analysis, and dynamic stimuli to test the proposed mechanisms.

## Figures and Tables

**Table 1:** Final sample sizes and exclusion distribution by group.

Analysis	Skilled (n)	Less-Skilled (n)	Total (N)	Excluded (Skilled)	Excluded (Less-Skilled)	χ^2^(1)	*p*
Recruited	50	51	101	—	—	—	—
Accuracy	44	45	89	6	6	0.001	>0.05
Decision time	44	45	89	6	6	0.001	>0.05
Eye-tracking	45	45	90	5	6	0.081	>0.05
EEG	36	39	75	14	12	0.264	>0.05

**Table 2:** Decision accuracy by skill and pattern (*N* = 89).

	Skilled (*M* ± *SD*)	Less-Skilled (*M* ± *SD*)
Structured	0.48 ± 0.13	0.40 ± 0.11
Unstructured	0.43 ± 0.09	0.41 ± 0.07

**Table 3:** Decision time by skill and pattern (*N* = 89).

	Skilled (*M* ± *SD*)	Less-Skilled (*M* ± *SD*)
Structured	1206.44 ± 347.21	1604.74 ± 488.28
Unstructured	1412.03 ± 362.87	1658.94 ± 509.61

**Table 4:** Pupil diameter by skill and pattern (*N* = 90).

	Skilled (*M* ± *SD*)	Less-Skilled (*M* ± *SD*)
Structured	2.96 ± 0.28	3.17 ± 0.45
Unstructured	2.83 ± 0.36	3.23 ± 0.46

**Table 5:** Fixation counts by AOI, skill, and pattern (*N* = 90).

	Total Fixation Counts (*M* ± *SD*)		Fixation on Teammate (*M* ± *SD*)		Fixation on Opponent (*M* ± *SD*)		Fixation on Ball (*M* ± *SD*)		Fixation on Open Space (*M* ± *SD*)	
	Skilled	Less-Skilled	Skilled	Less-Skilled	Skilled	Less-Skilled	Skilled	Less-Skilled	Skilled	Less-Skilled
Structured	6.09 ± 1.27	6.96 ± 0.88	1.10 ± 0.33	1.20 ± 0.28	0.68 ± 0.25	0.75 ± 0.17	0.17 ± 0.24	0.13 ± 0.07	4.17 ± 1.20	4.78 ± 0.79
Unstructured	6.51 ± 0.63	7.14 ± 0.78	1.04 ± 0.21	1.08 ± 0.27	0.71 ± 0.14	0.76 ± 0.17	0.18 ± 0.08	0.14 ± 0.06	4.58 ± 0.55	5.15 ± 0.65

**Table 6:** Fixation duration by AOI, skill, and pattern (*N* = 90).

	Total Fixation Duration (*M* ± *SD*)		Fixation on Teammate (*M* ± *SD*)		Fixation on Opponent (*M* ± *SD*)		Fixation on Ball (*M* ± *SD*)		Fixation on Open Space (*M* ± *SD*)	
	Skilled	Less-Skilled	Skilled	Less-Skilled	Skilled	Less-Skilled	Skilled	Less-Skilled	Skilled	Less-Skilled
Structured	1394.64 ± 287.36	1506.62 ± 335.64	247.22 ± 85.81	255.56 ± 90.78	152.35 ± 66.11	184.69 ± 73.72	27.13 ± 29.16	17.21 ± 12.98	967.94 ± 251.59	1049.16 ± 289.83
Unstructured	1626.61 ± 544.97	1886.89 ± 1314.43	286.10 ± 91.78	316.40 ± 110.61	131.57 ± 38.80	149.63 ± 39.22	33.52 ± 23.56	28.10 ± 19.41	1175.42 ± 493.77	1392.76 ± 483.77

## Data Availability

Data and materials are available on the Open Science Framework (OSF): https://osf.io/qny6u/overview?view_only=23e1f7651bd04b7797357502b430cf75 (accessed on 30 September 2026).

## References

[1] Martínez Ordóñez L., Ordóñez L.M., Villar J.R. (2017). Game theory and the decision-making process in military affairs. Military Operational Planning and Strategic Moves.

[2] Raab M., Bar-Eli M., Plessner H., Araújo D. (2019). The past, present and future of research on judgment and decision making in sport. Psychol. Sport Exerc..

[3] Araújo D., Hristovski R., Seifert L., Carvalho J., Davids K. (2019). Ecological cognition: Expert decision-making behaviour in sport. Int. Rev. Sport Exerc. Psychol..

[4] Vencúrik T., Bokůvka D., Nykodým J., Vacenovský P. (2020). Decision making of semi-professional female basketball players in competitive games. Sport and Quality of Life.

[5] Wurm M.F., Erigüç D.Y. (2025). Decoding the physics of observed actions in the human brain. eLife.

[6] Faubert J., Sidebottom L. (2012). Perceptual-cognitive training of athletes. J. Clin. Sport Psychol..

[7] Qiu F.H. (2019). A Study on the Brain Function and Structural Characteristics of Multi-Target Tracking Ability in High-Level Basketball Athletes. Master’s Thesis.

[8] Chu X.Y., Wang Z.J. (2024). Cognitive superiority of athletic sports expert and its formation mechanisms: A perspective from automaticity and abstraction. Adv. Psychol. Sci..

[9] Tenenbaum J.B., Kemp C., Griffiths T.L., Goodman N.D. (2011). How to grow a mind: Statistics, structure, and abstraction. Science.

[10] Ericsson K.A., Kintsch W. (1995). Long-term working memory. Psychol. Rev..

[11] Ericsson K.A., Krampe R.T., Tesch-Römer C. (1993). The role of deliberate practice in the acquisition of expert performance. Psychol. Rev..

[12] Kredel R., Hernandez J., Hossner E.J., Zahno S. (2023). Eye-tracking technology and the dynamics of natural gaze behavior in sports: An update 2016–2022. Front. Psychol..

[13] Roca A., Ford P.R., McRobert A.P., Williams A.M. (2011). Identifying the processes underpinning anticipation and decision-making in a dynamic time-constrained task. Cogn. Process..

[14] Klostermann A., Vater C., Kredel R., Hossner E.J. (2020). Perception and action in sports: On the functionality of foveal and peripheral vision. Front. Sports Act. Living.

[15] Vaeyens R., Lenoir M., Williams A.M., Mazyn L., Philippaerts R.M. (2007). The effects of task constraints on visual search behavior and decision-making skill in youth soccer players. J. Sport Exerc. Psychol..

[16] Allard F., Graham S., Paarsalu M.E. (1980). Perception in sport: Basketball. J. Sport Exerc. Psychol..

[17] Zhao Q., Wang Y., Lu Y., Luan M., Gao S., Li X., Zhou C. (2025). Computational bases of domain-specific action anticipation superiority in experts: Kinematic invariants mapping. Cognition.

[18] Chiu Y.K., Pan C.Y., Chen F.C., Tseng Y.T., Tsai C.L. (2020). Behavioral and cognitive electrophysiological differences in the executive functions of Taiwanese basketball players as a function of playing position. Brain Sci..

[19] Jin H., Xu G., Zhang J.X., Gao H., Ye Z., Wang P., Lin H., Mo L., Lin C.-D. (2011). Event-related potential effects of superior action anticipation in professional badminton players. Neurosci. Lett..

[20] Huang Z., Xue W., Ding A., Wei X. (2026). Electroencephalography in motor decision-making: A scoping review in athletes. Front. Psychol..

[21] Kanatschnig T., Schrapf N., Leitner L., Wood G., Körner C., Tilp M., Kober S.E. (2025). EEG theta and alpha oscillations during tactical decision-making: An examination of the neural efficiency hypothesis in volleyball. PLoS ONE.

[22] Zhao M., Meng H., Xu Z., Du F., Liu T., Li Y., Chen F. (2011). The neuromechanism underlying verbal analogical reasoning of metaphorical relations: An event-related potentials study. Brain Res..

[23] Zhao M., Xu Z., Liu T., Du F., Li Y., Chen F. (2012). The neural mechanism of language analogy reasoning: Evidence from ERP research. Acta Psychol. Sin..

[24] Du X.S., Wang A.P., Sun P. (2021). The effect of low and high avoidance-motivated negative emotions on analogical reasoning: Evidence from an ERP study. Psychol. Explor..

[25] Pegna A.J., Landis T., Khateb A. (2008). Electrophysiological evidence for early non-conscious processing of fearful facial expressions. Int. J. Psychophysiol..

[26] Feng Y. (2008). Research on the Cognitive Dominance Characteristics and Neural Mechanism of High-Level Foil Athletes. Master’s Thesis.

[27] Wang L. (2013). The Structural Features of Sport Information Mode and the Central Processing Mechanism. Doctoral Dissertation.

[28] Zhao H. (2010). Research on the cognitive characteristics and Neural Mechanism of Perception Prediction Process of Excellent Sanda Athletes. Master’s Thesis.

[29] Costa S., Berchicci M., Bianco V., Croce P., Di Russo F., Quinzi F., Bertollo M., Zappasodi F. (2023). Brain dynamics of visual anticipation during spatial occlusion tasks in expert tennis players. Psychol. Sport Exerc..

[30] Kalra J., Mittal P., Mittal N., Arora A., Tewari U., Chharia A., Upadhyay R., Kumar V., Longo L. (2023). How visual stimuli evoked P300 is transforming the brain-computer interface landscape: A PRISMA compliant systematic review. IEEE Trans. Neural Syst. Rehabil. Eng..

[31] Luck S.J., Handy T.C. (2005). Ten simple rules for designing and interpreting ERP experiments. Event-Related Potentials: A Methods Handbook.

[32] Williams A.M., Ford P.R. (2008). expertise and expert performance in sport. Int. Rev. Sport Exerc. Psychol..

[33] Yarrow K., Brown P., Krakauer J.W. (2009). Inside the brain of an elite athlete: The neural processes that support high achievement in sports. Nat. Rev. Neurosci..

[34] Meng F.Y., Geng J.X., Li A.M. (2022). The effect of sports experience on unconscious processing of table tennis players: Evidence from ERP and behavioral experiments. China Sport Sci. Technol..

[35] Wang Y.Y., Lu Y.Z., Yang T.T., Gu N., Zhou C.L. (2020). The effect of conceptual experience on action processing of table tennis players: An ERP study. J. Shanghai Univ. Sport.

[36] Beatty J., Lucero-Wagoner B., Cacioppo J.T., Tassinary L.G., Berntson G.G. (2000). The pupillary system. Handbook of Psychophysiology.

[37] Donchin E., Coles M.G. (1988). Is the P300 component a manifestation of context updating?. Behav. Brain Sci..

[38] Diederich A., Busemeyer J.R. (2006). Modeling the effects of payoff on response bias in a perceptual discrimination task: Bound-change, drift-rate-change, or two-stage processing hypothesis. Percept. Psychophys..

[39] Liu J., Zhang C., Zhu Y., Liu Y., Parviainen T. (2020). Dissociable effects of reward on P300 and EEG spectra under conditions of high vs. low vigilance during a selective visual attention task. Front. Hum. Neurosci..

